# Body dysmorphic disorder treatment: about a clinical case

**DOI:** 10.1192/j.eurpsy.2022.994

**Published:** 2022-09-01

**Authors:** R. André, F. Azevedo, M. Gonçalves, J. Romão, R. Saraiva, M. Croca, M. Abreu

**Affiliations:** 1 Centro Hospitalar Universitário Lisboa Norte, Psychiatry, Lisboa, Portugal; 2 Centro Hospitalar de Lisboa Ocidental, Psychiatry, oeiras, Portugal

**Keywords:** Body Dysmorphic Disorder, Treatment, dysmorphofobia, surgery

## Abstract

**Introduction:**

Body dysmorphic disorder (BDD) is a relatively common disorder characterized by a preoccupation with non-existent or slight defects in appearance. It was first described in 1886 by Morselli as dysmorphophobia.

**Objectives:**

This work reviews the current available data on BDD and its treatment options and describes a clinical case that reports an improvement in symptomatology after surgery.

**Methods:**

Non-systematic review of the literature with selection of scientific articles published in the past 10 years; by searching Pubmed and Medscape databases using the combination of MeSH descriptors. The following MeSH terms were used: “body dysmorphic disorder”, “dysmorphophobia”. Clinical file consultation.

**Results:**

The usual treatment involves a combination of psychotherapy and pharmacotherapy. Antidepressant medication, mainly selective serotonin reuptake inhibitors (SSRIs) have been used. If the symptoms do not improve, a different SSRI can be considered or clomipramine, venlafaxine or second-generation antipsychotics can be useful.

**Conclusions:**

The role of surgery remains controversial, several studies indicating that the symptoms typically worsen after an aesthetic procedure because the preoccupation shifts to a different body area. However a recent study reported 32 of the 41 patients that underwent surgery were highly satisfied with the outcome. In our clinical case, our patient, a 20-year-old female with non-delusional dysmorphic ideas about her nose initiated treatment with paroxetine with poor response and was, against medical opinion, submitted to a rhinoplasty. Three weeks after the surgery there was an improvement in preoccupation about her nose. More research should be made to clarify the role of surgery in this disorder that often lacks adequate therapeutical response.

**Disclosure:**

No significant relationships.

